# A Clean-Label Formulation of Fortified Yogurt Based on Rhododendron Flower Powder as a Functional Ingredient

**DOI:** 10.3390/foods12234365

**Published:** 2023-12-04

**Authors:** Alina Narcisa Postolache, Ionuț Dumitru Veleșcu, Florina Stoica, Ioana Cristina Crivei, Vlad Nicolae Arsenoaia, Marius Giorgi Usturoi, Cristina Gabriela Constantinescu (Pop), Florin Daniel Lipșa, Gabriela Frunză, Daniel Simeanu, Roxana Nicoleta Rațu

**Affiliations:** 1 Research and Development Station for Cattle Breeding Dancu, 707252 Iasi, Romania; narcisa.postolache@gmail.com; 2 Department of Food Technologies, Faculty of Agriculture, ”Ion Ionescu de la Brad” University of Life Sciences, 3 Mihail Sadoveanu Alley, 700489 Iasi, Romania; ionut.velescu@uaiasi.ro (I.D.V.); flipsa@uaiasi.ro (F.D.L.); frunza.gabriela@uaiasi.ro (G.F.); 3 Department of Pedotechnics, Faculty of Agriculture, ”Ion Ionescu de la Brad” University of Life Sciences, 3 Mihail Sadoveanu Alley, 700489 Iasi, Romania; florina.stoica@uaiasi.ro (F.S.); vnarsenoaia@uaiasi.ro (V.N.A.); 4 Department of Public Health, Faculty of Veterinary Medicine, “Ion Ionescu de la Brad” University of Life Sciences, 6 Mihail Sadoveanu Alley, 700449 Iasi, Romania; i.crivei@uaiasi.ro; 5 Department of Animal Resources and Technology, Faculty of Food and Animal Sciences, “Ion Ionescu de la Brad” University of Life Sciences, 8 Mihail Sadoveanu Alley, 700489 Iasi, Romania; 6 Department of Food Technologies, Safety of Food Production and the Environment, Faculty of Food Engneering, Stefan cel Mare University of Suceava, 13 University Street, 720229 Suceava, Romania; gabriela.constantinescu@fia.usv.ro; 7 Department of Control, Expertise and Services, Faculty of Food and Animal Sciences, “Ion Ionescu de la Brad” University of Life Sciences, 8 Mihail Sadoveanu Alley, 700489 Iasi, Romania; dsimeanu@uaiasi.ro

**Keywords:** rhododendron flowers, phytochemicals, antioxidant, functional ingredient, fortified yogurt

## Abstract

The world-wide-dispersed Rhododendron is a tiny, evergreen plant with vivid red or pale pink blossoms that is a member of the *Ericaceae* family and is well-known for its stunning flowers. To improve yogurt’s nutritional profile and sensory qualities, this study investigates an innovative application of Rhododendron flower powder (RFP). The potential health benefits of Rhododendron flowers, which are a rich source of bioactive compounds such as polyphenols and antioxidants, have attracted attention. Consequently, the physicochemical, phytochemical, and sensory qualities of fortifying yogurt with RFP at various concentrations were studied. The results showed that the texture and color of the yogurt were highly influenced by the addition of RFP. The addition of this functional ingredient also resulted in a significant increase in the yogurt’s polyphenol content and antioxidant capacity. These findings demonstrate the suitability of RFP in yogurt formulations as a functional food ingredient, being a good source of phenolics.

## 1. Introduction

Nowadays, consumers are more aware and concerned about the components in the food they purchase, preferring foods made from natural ingredients or derived from natural sources, avoiding those products with added artificial additives [1]. Furthermore, the increasing demand for nutraceutical products has led to the emergence of novel types of healthy food resources, such as fortified foods and natural food products containing natural constituents. Studies have revealed that some phenolic compounds can be added to various food matrixes to fortify them by increasing their nutritional value and complying with changing market trends [2,3].

Rhododendron parts are rich in bioactive phenolic compounds with strong antioxidant properties. Its young leaves were used to treat headaches. The flowers contain high levels of secondary metabolites like rutin, coumaric acid, anthocyanins (delphinidin, cyanidin, malvidin 3-O-arabinoside-5-O-glucosides), and flavonoids (quercetin-3-rhamnoside) [4,5,6]. The genus’s diverse metabolites, including anthocyanins, flavonoids, and phenolic acids, are noted for their therapeutic qualities and potential in nutraceuticals [7].

Phenolic compounds have a wide range of biological characteristics, which makes them very appealing to various sectors. Indeed, research has revealed that these substances exhibit biological attributes, including antibacterial, antiallergic, anticancer, antiaging, and inhibitory effects towards enzymes like α-amylase and β-glucosidase. As a method of preventing disorders like type 2 diabetes, the inhibition of these enzymes is crucial for delaying an increase in blood glucose levels [8]. Nevertheless, it is the antioxidative properties demonstrated by these compounds that made the Rhododendron plant especially desirable. Phenols can operate as natural antioxidants by reducing oxidative stress, preventing chain auto-oxidation reactions, and reducing the production of free radicals [9].

With a few exceptions, researchers and processors in the food and medicinal sectors have not yet taken advantage of the flower’s limited availability. Recently, food manufacturers have become interested in using Rhododendron flowers to provide beneficial food products. Rhododendron flower juice is thought to have considerable medical potential in addition to being a delightful beverage. [5]. Rhododendron honey is reportedly used as medication and has a history of treating several conditions. The active compounds are andromedotoxin and grayanotoxin [10]. For the preparation of ready-to-serve drinks and squash, researchers used flowers from the Madhuca indica and Rhododendron arboretum [11,12]. Rhododendron (*Rhododendron arboreum* Sm.) flower petals were used to obtain functionally enriched squash [13]. They claimed that anthocyanin, phenols, and ascorbic acid are among the several antioxidant substances found in Rhododendron blooms. Honey-based varieties were shown to have the richest antioxidant components when compared to Rhododendron squash made with sugar.

Milk and dairy products are essential foods for human nutrition. Therefore, the shelf life of these items depends on their oxidative stability because these foods are prone to oxidation, which typically results in the formation of unpleasant flavors and smells and reduces the nutritional characteristics [14]. Lactic acid bacteria (LAB) fermentation produces yogurt, a commonly consumed coagulated milk product and a health-promoting food [15]. It has notable concentrations of nutritious ingredients, including proteins, peptides, minerals (such as calcium, phosphorus, and potassium), and vitamins (including vitamin D, B12, and riboflavin). Despite its positive health effects, yogurt is generally not regarded as a significant source of bioactive compounds [16]. Nowadays, milk products fortified with natural elements are among the best ways to improve overall food intake with the least negative side effects. [16]. As a result, fortifying yogurt with bioactive plant resources—such as common purslane (*Portulaca oleracea*) extract [17], bilberry (*Vaccinium myrtillus* L.) residues [18], and encapsulated carrot waste extract [19]—has drawn more attention in an effort to enhance the nutritional qualities and health-promoting advantages of plain yogurt. It is important to note that adding plant extracts to yogurt can enhance its concentration of bioactive components and cause the release of bioactive peptides, which increases the yogurt’s antioxidant potential. It is important to note that adding plant extracts to yogurt can enhance its concentration of bioactive components and cause the release of bioactive peptides, which increases the yogurt’s antioxidant potential. [20].

The process of fortifying the milk base is a crucial step that significantly improves the functional and nutritional characteristics. However, to our knowledge, no study focused on the potential of rhododendron flowers in functional yogurt.

Several artificial additives (tartrazine, ponceau 4R red 40, Bordeaux S, erythrosine, indigotin, vanilla flavor, sodium nitrate, and potassium sorbate) [21,22] have been employed in the agrifood industry to enhance color and flavor, and to prolong the shelf life of yogurt. However, numerous studies have demonstrated a link between the overuse of artificial food additives and unfavorable respiratory, dermatological, gastrointestinal, and neurological effects [23,24]. Researchers have, therefore, developed additives from natural sources that would be suitable for use in dairy products as a response to the requirement to utilize safe additives [25,26].

The use of RFP in the composition of yogurt can improve the product’s nutritional value, thereby enhancing the overall quality of life. Hence, the aim of this study was to extract the bioactive compounds from Rhododendron powder and evaluate the extract for its total anthocyanins content, flavonoids, phenolic compounds, and antioxidant activity. This study also assessed the impact of RFP on the physicochemical, phytochemicals, antioxidants, color, texture, and sensory characteristics of yogurts enriched with RFP. Moreover, the research aimed to determine whether phenolic-enriched powders could be a sustainable food ingredient for producing more nutrient-dense foods.

## 2. Materials and Methods

### 2.1. Materials

A total of 125 L of milk was extracted from the storage tank of a dairy cattle farm located in the northeastern part of Romania. The milk was transferred in proper conditions (maximum 4 °C), using a thermally regulated refrigerating tank, to the dairy products processing laboratory within the University of Life Sciences, Iasi County. A sample was obtained from the initial volume to assess the physical-chemical parameters of the milk used in the experiment. The sample was carefully transferred to the laboratory under optimal conditions (6 °C), using a food-grade stainless steel container, and stored thereafter at a temperature of a maximum 4 °C for 24 h. Following a rigorous homogenization process, the physical-chemical parameters, such as moisture, fat, protein ash content, and pH, were determined using the techniques specified by the AOAC [24]. The reagents used for determinations were as follows: ethanol, methanol, 2,2-diphenyl-1-picrylhydrazyl (DPPH), 6-hydroxy-2,5,7,8-tetramethylchroman 2-carboxylic acid (Trolox), Folin-Ciocalteu reagent, gallic acid, sodium carbonate, sodium acetate, sodium nitrite, sodium hydroxide, potassium chloride, and aluminum chloride, purchased from Sigma Aldrich (Steinheim am Albuch, Germany).

### 2.2. Rhododendron Flowers Powder Preparation

*Rhododendron myrtifolium* samples were collected across northeastern Romania. Samples were manually removed to collect the flowers. Rhododendron flowers were freeze-dried under −42 °C and a pressure of 0.10 mBar (BIOBASE BK-FD10T Freeze Dryer, Jinan, China) for 48 h up to 98% dry weight. Dried Rhododendron flowers were ground (MC 12 equipment, Stephan, Frauenberg, Germany) and passed through various sizes of sieves to acquire powder with appropriate particle sizes (0.85 mm) and stored in glass containers with lids at room temperature until analysis. The final form of powder underwent decontamination through sterilization using a UV lamp.

### 2.3. Extraction of Phytochemicals from Rhododendron Flowers Powder (RFP)

Ultrasound-assisted extraction was used to extract the phytochemicals from Rhododendron powder [27]. In brief, 1.0 g of Rhododendron powder was mixed with 10 mL of 70% ethanol acidified with glacial acetic acid (ratio 9:1, *v*/*v*) and subjected to a sonication water bath (Elmasonic S 180 H, Singen am Hohentwiel, Germany) for 40 min at +40 °C and a frequency of 40 kHz. After recovering the resulting crude extract, it was then centrifuged (Centurion Scientific K3 series, Carlsbard, CA, USA) for 5 min at 7000 rpm and 10 °C. The supernatant was collected after separation, and the residue was extracted two more times using 10 mL of 70% ethanol. Moreover, the supernatant was collected and concentrated in a rotary evaporator at 40 °C using IKA RV 10 control (Staufen, Germany). The concentrated extracts were then phytochemically analyzed.

### 2.4. The Quantification of Anthocyanins, Phenolic Compounds, and Evaluation of Antioxidant Potential of Rhododendron Flowers Powder (RFP)

#### 2.4.1. Total Anthocyanin Content

A modified pH differential approach was used to determine the total monomeric anthocyanins content [28]. The samples were diluted (D = 1:10) before analysis. The absorbance of the diluted extracts was then determined at two different wavelengths: 520 nm and 700 nm (UV–vis Spectrophotometer, Analytik Jena-Specord 210 Plus, Jena, Germany), using 200 µL of vegetable extract and 800 µL of a buffer solution with a pH of 1.0/4.5. The results were expressed in milligrams of cyanidin-3-glucoside (C3G) per gram of dry weight (DW) and were calculated using the following Equation (1):

Total anthocyanin content (mg/g) = (A × MW × DF × Vt)/(M × ε × 1)(1)

where A is the difference between (A520–A700) at pH 1.0 and (A520–A700) at pH 4.5, MW is the molecular weight (449.2 g/mol) of cyanidin-3-glucoside, DF is the dilution factor, Vt is the total volume (mL), M is the weight of sample (g), ε is the molar extinction coefficient (26,900) of cyanidin-3-glucoside, and l is the path length.

#### 2.4.2. Total Flavonoid Content

Using the technique described by Stoica et al. [29], the total flavonoid content of Rhododendron powder extract was assessed using the aluminum chloride method. Briefly, 2 mL of distilled water and 0.50 mL of extract solution were mixed with 0.15 mL of NaNO_2_ 5% solution. After adding 0.15 mL of a 10% AlCl_3_ solution and a waiting time of 5 min, the mixture was allowed to rest for an additional 6 min. After 1 mL of a 1 M NaOH solution was added, the absorbance at 510 nm was measured (UV–vis Spectrophotometer, Analytik Jena-Specord 210 Plus, Germany). The total flavonoid concentration was calculated using the linear regression equation of the calibration curve (R^2^ = 0.9978), which was derived using quercetin as a standard. The results were expressed as milligrams of quercetin equivalents (mg QE) per gram of dry weight (DW).

#### 2.4.3. Total Polyphenolic Content

The total polyphenolic content was determined using the modified technique developed by Dewanto et al. [30]. In brief, 200 µL of extract with 15.8 mL of ultrapure water and 1 mL of Folin–Ciocalteu reagent were mixed. After 10 min, 3 mL of 20% Na_2_CO_3_ was added, and the mixture was kept at +25 °C in dark conditions for 60 min. The mixture’s absorbance was measured at a 765 nm wavelength (UV–vis Spectrophotometer, Analytik Jena-Specord 210 Plus, Germany). Gallic acid equivalents (mg GAE) per gram of dry weight (DW) were used to express the results. The standard curve’s Gallic acid concentration ranged from 10 to 100 ppm, and the resulting equation was y = 1.6991x − 0.0256.

#### 2.4.4. Antioxidant Activity (DPPH)

The antioxidant activity using 2,2-diphenyl-1-picrylhydrazyl (DPPH) of Rhododendron powder extract was assessed by Stoica et al. [29]. Thus, a mixture was created by combining a 100 µL extract with 3.9 mL of a 0.1 M DPPH solution (Af). The solution was kept at +25 °C for 90 min in dark conditions. The mixture’s absorbance was measured at a 515 nm wavelength (UV–vis Spectrophotometer, Analytik Jena-Specord 210 Plus, Germany). The control was obtained by mixing 3.9 mL of 0.1 M DPPH solution with 100 µL of methanol (A0), and the absorbance mixture was likewise measured. The results were presented as µmol Trolox equivalents (TE)/g DW. The standard curve’s Trolox content ranged from 10 to 100 ppm, and the resulting equation was y = 0.45x + 0.0075.

### 2.5. Preparation of Yogurt Supplemented with RFP

**Figure 1:**
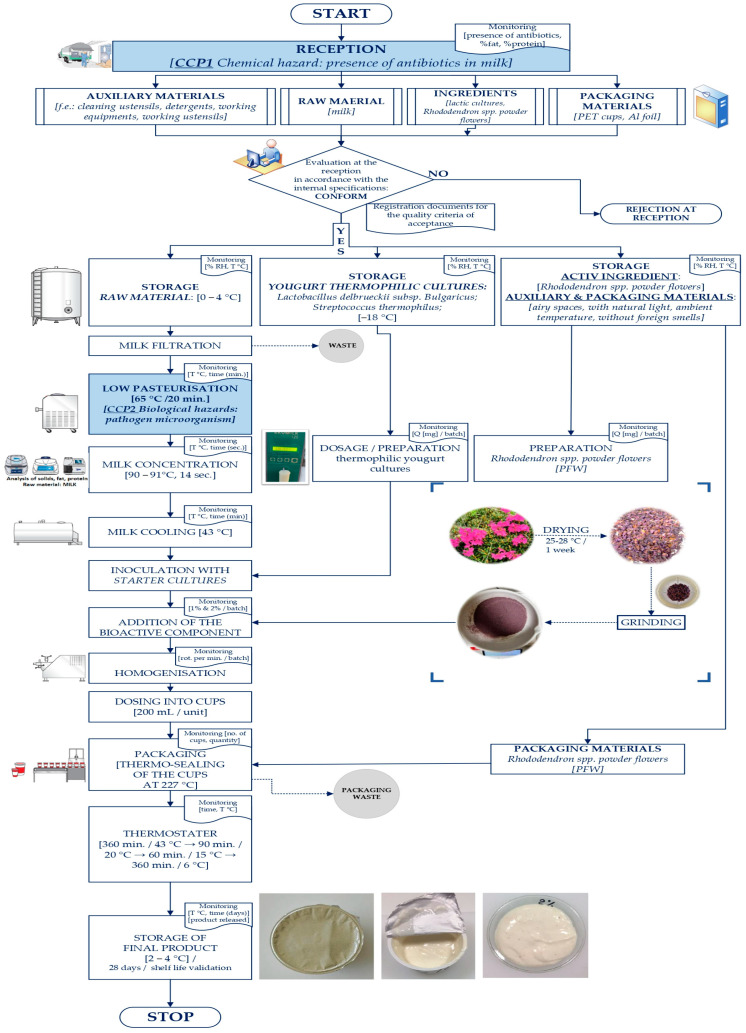
Functional yogurt prototype processing flow diagram.

Our clean-label yogurt was manufactured in accordance with the flow chart specified in Figure 1, under the GHP and GMP Guidelines, which comply with Codex Alimentarius rules of food safety through an efficiently implemented HACCP system, updated in May 2023 [31].

The technological process of yogurt preparation with Rhododendron powder begins with the reception stage of the raw material and ingredients (cow’s milk, lactic cultures, RFP) and auxiliary materials (Figure 1). From the standpoint of food safety, the raw material milk reception stage is the critical control point no. 1 (CCP) that, through measures taken at reception (qualitative rapid tests), eliminates the presence of antibiotic traces in milk [32]. Also, at this stage, the raw milk is analyzed in terms of its lipid and protein content. Following the evaluation at the reception, according to the internal specifications, the registration documents for the acceptance quality criteria are maintained.

The storage of raw material and auxiliary materials is carried out as follows: the milk is stored in isothermal tanks, the temperature being monitored (0–4 °C); lactic cultures for yogurt are stored at a temperature of −18 °C, monitoring the temperature and humidity; the active ingredient (RFP 1 and 2%); auxiliary and packaging materials are kept in ventilated spaces, with natural light, without foreign odors at ambient temperature; the temperature and relative humidity of the air is monitored for product compliance.

After filtering, the milk is subjected to the low-pasteurization process. According to the Codex Alimentarius decision tree, milk pasteurization is the second control critical point (CCP2), where the occurrence of microbiological risk regarding pathogenic microorganisms in milk is prevented. In this context, the critical limits of the pasteurization process were confirmed based on legislation and registered and maintained during the thermal treatment process (pasteurization temperature and time of raw milk: 65 °C/20 min). The milk was concentrated at a temperature of 90–91 °C for 14 s. The effectiveness of the concentration process is achieved by analyzing the fat and protein content as well as the total dry substance. Subsequently, the milk was cooled while the temperature was monitored, which was maintained at 43 °C.

The inoculation of milk with lactic starter cultures was the next step in the technological process after they had previously been dosed and prepared. The quantity of lactic starter cultures per batch was also monitored (mg/batch). The next step in the technological procedure was adding the bioactive component and monitoring its level (1% and 2%/batch). The preparation stage for the bioactive component consisted of obtaining Rhododendron powder (by drying the flowers, followed by grinding).

The yogurt was dosed into PET glasses with a capacity of 250 mL, followed by their thermo-welding at 227 °C. The quantity and number of glasses were recorded.

The thermostating step process involved maintaining the yogurt-filled glasses at different temperatures for a certain timeframe. During this stage, the thermostat time and temperature were monitored as follows: 360 min/43 °C; 90 min/20 °C; 60 min/15 °C; 360 min/6 °C. Afterward, the yogurt was stored at a temperature of 2–4 °C for 28 days. A shelf life of 28 days for the yogurt formula was established and validated using the control yogurt as a basis. In this stage, the storage temperature, the storage time, and the product delivery date were monitored. All indicated parameters complied with the established product requirements.

### 2.6. Physicochemical Characterization of Yogurts Supplemented with RFP

The moisture content, total protein, fat, ash, and pH of the samples were analyzed using the methodology outlined by the Association of Official Analytical Chemists (AOAC). The methodology outlined by Mbaeyi-Nwaoha et al. [33] was employed to ascertain the crude fiber content. The determination of the total energy value was conducted using the Atwater method, as outlined in the study by Ezeonu et al. [34]. This approach implies multiplying the percentage of carbohydrate content by 4%, the percentage of protein content by 4%, and the percentage of fat content by 9%. The measurement of energy was conducted using kilocalories per kilogram (Kcal/100 g).

Energy value = (%CP × 4) + (%CFT × 9) + (%CHO × 4)(2)

where: %CP–percentage crude protein; %CFT–percentage crude fat; %CF = percentage crude fiber; %CHO = percentage carbohydrate.

Igbabul et al. [35] provided the mathematical formula used to calculate carbohydrates (CHO).

CHO = 100 − % (ash + protein + fat + crude fiber + moisture)(3)

### 2.7. Characterization of Phytochemicals and Antioxidant Activity of Yogurts Supplemented with RFP

The method of Pan et al. [36] was used to determine phytochemicals in the yogurt samples. To fully precipitate the remaining protein, 10 g of yogurt water extracts were combined with 15 mL of acidified methanol (containing 0.1% HCl) and put at −20 °C for one hour. The samples were centrifuged before bioactive compounds determination at 14,000 rpm for 10 min at 4 °C. The resulting supernatant, after centrifugation, was used for the phytochemical determination. The total anthocyanins, total polyphenolic contents, total flavonoid contents, and antioxidant activity of yogurt supplemented with RFP were assessed using the techniques described in Section 2.4.1, Section 2.4.2, Section 2.4.3 and Section 2.4.4.

### 2.8. Colorimetric Analysis of Yogurt Supplemented with RFP

A MINOLTA Chroma Meter CR-410 (Konica Minolta, Osaka, Japan) was used to analyze the colors of the dried RFP and yogurt samples. The sample color values are represented by the following symbols: L* (whiteness/darkness), a* (redness/greenness), and b* (yellowness/blueness). For each sample, there were three replicates. As well as the Chroma value of $\sqrt{{{(a}^{*})}^{2}+{{(b}^{*})}^{2}}$, and Hue angle of arctan(b*/a*) to represent the saturation and shade of the color, respectively [37].

### 2.9. Texture Profile Analysis of Yogurt Supplemented with RFP

Using a Brookfield CT3 Texture Analyzer (Brookfield Ametek, Middleboro, MA, USA) and a 38.1 mm diameter acrylic cylinder, the samples were subjected to a double penetration test to determine the texture profile. The test parameters were as follows: trigger load 0.067 N, load cell 9.8 N, target distance 15 mm, and penetration speed 1 mm/s. All measurements were realized in a controlled room at 20 °C. The deformation-stress response was processed using TexturePro CT V1.5 software, and textural characteristics were calculated, such as hardness, adhesiveness, cohesiveness, springiness, and gumminess [38]. For each sample, three replications were carried out.

### 2.10. Sensorial Evaluation of Yogurt Supplemented with RFP

The sensorial evaluation of yogurt with and without RFP was performed by 20 untrained panelists with regular yogurt consumption (more than once a month) between 18 and 39 years old, including 40% males and 60% females were randomly recruited. They were informed of the study’s overall objective and the necessary procedures for handling personal data. On a 9-point hedonic scale, panelists were asked to evaluate the appearance, color, aroma, texture, taste, odor, aftertaste, overall acceptability, consistency, smell, and general acceptability of the fortified yogurts (1 = extremely dislike; 9 = extremely like). The sensory evaluation space was maintained in a suitably air-conditioned setting, with the temperature in the booths regulated at around 25 °C. The samples were displayed in a randomized order. Panelists were asked to rinse their mouths thoroughly with water after each sample examination.

### 2.11. Statistical Analysis

Table data were presented as mean and standard deviation. Triplicate analyses of each sample were performed. The disparity between the averages was assessed using the GraphPad Prism 9.4.1 software (Graph Pad Ltd., Palo Alto, CA, USA) for statistical analysis. One-way ANOVA (analysis of variance) was used to assess the statistical data; *p* < 0.05 was chosen as the significance level, and the Tukey test was performed.

## 3. Results

### 3.1. The Phytochemical Characterization of RFP Extract

The phytochemical content, antioxidant activity, and color parameters of the RFP extract were determined, and the results are shown in Table 1.

The ultrasound-assisted method applied in the present study allowed us to obtain a bioactive-enriched extract containing total anthocyanins of 1.05 ± 0.24 mg C3G/g DW, total flavonoids of 8.58 ± 0.19 mg CE/g DW, with a total polyphenolic content of 21.46 ± 0.63 mg GAE/g DW. The extract showed a DPPH radical scavenging capacity of 16.89 ± 0.22 µmol TE/g DW. The parameter a* positive value demonstrates a tendency towards producing a red color that can be attributed to the higher anthocyanin content. The blue-to-yellow intensity is represented by the parameter b*, with a positive value indicating a trend towards yellow shades in the powder. All data were plotted in the first quadrant (+a*, +b*) based on the results for the values of a* and b*, indicating a propensity toward yellow and red, which is characteristic of anthocyanins. Chroma, which depicts the intensity and saturation of a color, was 12.94 ± 0.08. Given that the hue angle was less than 10°, the value indicated the redness of the powder and was proportionate to the received color.

### 3.2. Physicochemical Analysis of Yogurt Supplemented with RFP

The results of the physicochemical composition of the RFP are summarized in Table 2. The addition of 1.0% and 2.0% of RFP increased the fat, total protein, ash content, crude fiber, CHO, and energy values of the enriched yogurts compared with the control on the same day of storage. It can be observed that the pH values of yogurts supplemented with RFP differ significantly (*p* < 0.05), when compared to the control at the same day of storage. All formulated yogurts significantly decreased in moisture and pH when compared to the control yogurt.

Furthermore, it is noticeable that the fat content of the formulated yogurt decreased gradually when the concentrations of RFP increased proportionally on day 1, day 14, and after 28 days of storage. There is no significant difference in moisture, total protein, ash, and CHO content between the control and fortified yogurt samples (*p* > 0.05) at the end of storage.

### 3.3. Phytochemical Content and Antioxidant Activity of Yogurt Supplemented with RFP

Table 3 shows the phytochemical content and antioxidant activity of fortified yogurts and their stability during 28 days of storage.

Compared with the control, the addition of RFP dose-dependently increased the phytochemicals (anthocyanins, flavonoids, polyphenols) and the free radical scavenging activities of RFP-supplemented yogurt products (*p* < 0.05). During the storage, the phytochemicals values and DPPH values gradually decreased. All RFP-supplemented yogurts showed the lowest phytochemicals and antioxidant activity on the 28th day of storage. There was a high correlation between the polyphenolic compounds and antioxidant activity. The antioxidant activity was reduced on day 28. Similar patterns of change were also documented in other investigations [39,40]. According to Tseng and Zhao [39] and Yuksel et al. [40], the degradation of phenolics by polyphenol oxidase of LAB and protein–polyphenol interaction may be responsible for the loss of yogurt’s antioxidant capacity.

**Figure 2:**
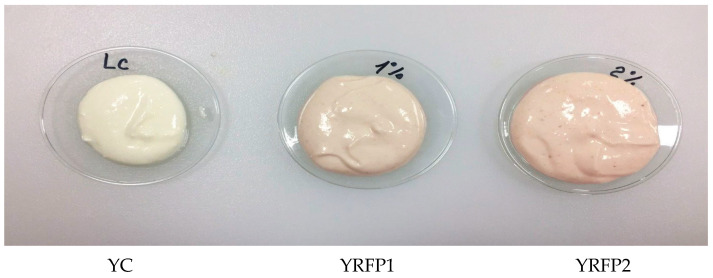
Images of the yogurt without RFP, control (YC); yogurt with 1% RFP (YRFP1); yogurt with 2% RFP (YRFP2).

Figure 2 depicts the appearance of the yogurts after blending with the RFP at concentrations of 1.0 and 2.0 g/100 g (*w*/*w*). The powder was evenly distributed throughout the product without any stains or sedimentation. The resulting hue intensified as expected, with rising RFP concentrations. As a result, the RFP was able to provide the yogurt’s color.

### 3.4. Textural Profile Analysis

Fermented dairy products’ structure and protein network organization influence their textural characteristics. Table 4 presents the findings of the texture profile analysis. The addition of RFP significantly affected all textural characteristics of the supplemented yogurts. Hardness is the most essential characteristic in determining yogurt texture. It evaluates how hard the yogurt is and is viewed as the force required to produce a particular deformation. The hardness of yogurt supplemented with RFP increased with the increase in concentration of the added powder. Increasing the hardness of the samples may be attributed to dietary fiber from rhododendron powder absorbing more moisture due to its elevated water-holding capacity [41]. The RFP-supplemented yogurts exhibited lower springiness and higher hardness, gumminess, cohesiveness, and adhesiveness compared with the control sample. All the texture parameters of RFP-supplemented yogurt increased gradually during the 28 days of cold storage.

### 3.5. Colorimetric Analysis

Acceptance of food is influenced by a variety of factors, such as color, flavor, and newly discovered health advantages. An important physical indicator of a dairy’s quality is color. Table 5 lists the findings from measurements of the samples’ chroma, hue angle, and color parameters L*, a*, and b*.

The control sample presented a significant maximum overall lightness (96.97) compared with the other supplemented yogurts. With the increase in RFP addition, L* values decreased statistically (*p* < 0.05), indicating that yogurt supplemented with RFP was darker than the control yogurt. The redness and yellowness significantly (*p* < 0.05) increased, parallel with the increase in RFP supplementation. This may be attributed to the natural pigments originating from Rhododendron powder. Also, the decreased values of L* (lightness) together with the increased values of a* (redness) and b* (yellowness) were identified during 28 days of storage in yogurt supplemented with RFP, as compared to the control. Chroma, a measure of the color’s intensity and saturation, exhibited the same pattern as the parameter b*, indicating that the yellow hue was the most descriptive in determining the sample’s color. A hue angle of 0° or 360° indicates a red hue for the studied samples. The supplemented yogurts’ measurements of L*, a*, and b* values changed during storage, becoming slightly darker (L < 0), redder (a > 0), and more yellow (b > 0).

### 3.6. Sensorial Evaluation

**Figure 3:**
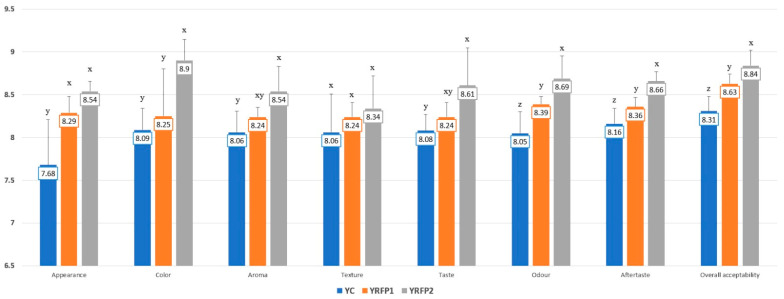
Sensory evaluation scores of control and fortified yogurts (averages with different letters “x”, “y”, “z” in the columns signify statistically significant differences (*p* < 0.05)).

The sensory analysis of the formulated yogurts was acquired using a 9-point hedonic scale. The analysis evaluated sensorial attributes such as appearance, color, aroma, texture, taste, odor, aftertaste, and overall acceptability. Figure 3 presents the average results from the sensory evaluation. According to the findings, adding 1% and 2% (*w*/*w*) RFP to yogurt showed good overall consumer acceptability for most of the characteristics evaluated. RFP to yogurt showed good overall consumer acceptability for most of the characteristics evaluated, especially for the yogurt with 2% RFP (YRFP2). With an increase in RPE concentration, the hedonic scores of overall acceptability rose. Of all the yogurts, those with 2% RFP added had the highest sensory scores for overall acceptability attributes. This suggests that adding RFP to yogurts can enhance their sensory qualities.

The sensory evaluation scores were considerably (*p* < 0.05) different between the fortified and control yogurt after adding various amounts of RFP. The addition of RFP up to 2% in yogurt received a “like very much (8)” liking score on appearance, odor, aroma, texture, taste, aftertaste, and overall acceptability in comparison with control yogurts by the panelists (Figure 3). The samples with the highest ratings across all eight attributes were YRFP2 yogurts with 2% RFP.

## 4. Discussion

The Rhododendron flower extract had a high content of polyphenols and remarkable antioxidant activity. RFP is noteworthy for its coloring ability and its antioxidant activity and can be used as a natural ingredient in food products. The results attained are consistent with the information presented in other investigations. Therefore, Jing et al. [42] determined the total flavonoid content and total phenolics content in the aerial part of R. anthopogonoides, and the values were 231.37 ± 4.56 mg rutin equivalents/g extract and 165.00 ± 19.39 mg GAE/g extract. Also, the DPPH radical scavenging activity was 127.46 ± 0.95 µmol TE/g. Rafi et al. [43] reported a lower content of total anthocyanins (1.04 ± 0.03 mg/100 g DW), and higher total phenolics (48.11 ± 0.79 mg GAE/g DW), total flavonoids (8.92 ± 0.34 mg QE/g DW), and antioxidant activity (29.00 ± 0.20 µmol TE/g dried powder) in R. jasminiflorum leaves as compared with our findings. Due to variations in raw material phytochemical variability, raw material fractions, extraction methods, and environment, all of these authors reported different outcomes for the phytochemical composition of the rhododendron extract. Color values of RFP show that RFP can be classified as red and yellow source of pigments. This could be attributed to the presence of phenolic compounds, especially anthocyanins. Since the hue angle was less than 10, the hue angle value revealed the redness of the RFP extract and was proportionate to the received color [44].

Yogurt’s nutritional profile will change slightly when natural functional components like those from fruits, vegetables, grains, and other foods are added [45,46]. The incorporation of RFP into yogurt significantly improved its chemical composition compared to the control. Moreover, adding 2% powder to yogurt increased the fat, total protein, ash, crude fiber, and CHO. These results may be due to Rhododendron powder having a high amount of carbohydrates and proteins [47]. These findings comply with other studies. After one day of storage, the amount of protein, carbs, fat, total solids, and ash in stirred-type yogurt made from commercially homogenized and pasteurized milk that was fortified with 1% skimmed milk powder was 3.98, 5.35, 3.40, 13.99, and 0.70% [46]. These components were reduced after the yogurt was kept for 7 to 21 days at 4 °C. This could be because yogurt contains microorganisms that depend on protein, carbs, and fat for growth and metabolism. Yogurt’s excessive acidification is the term used to describe the pH drop that occurs during storage [48].

Increasing RFP concentration, from 1 to 2%, increased the anthocyanins, flavonoids, polyphenols, and in vitro antioxidant activity of supplemented yogurts, thus indicating a dose-dependence effect. These findings are consistent with those of Demirkol et al. [49], who found that yogurt containing grape pomace (1 to 5 g/100 g milk) increased the polyphenols (20 to 52 mg/100 g yogurt) and DPPH value (263 to 1100 mg/mL for 50% inhibition of the radical). Kennas et al. [50] reported on the positive impacts of additional fruit by-products on yogurt polyphenols and found a favorable association between yogurt polyphenols levels and the amounts of added pomegranate peel. Data in Table 3 also showed that the phytochemicals of control and supplemented yogurt samples gradually decreased during refrigerated storage, which was consistent with other yogurts containing various fruit extracts [51,52]. A slow breakdown of phenolic compounds by LAB and the production of aromatic acids, including asphenyl propionic, acetic, and benzoic acids, during refrigeration could be responsible for the reduction in phenolic compounds. The hydrolysis of polyphenolic compounds by probiotic bacteria found in yogurt was described by Muniandy et al. [53]. In yogurts enriched with banana peel flour, Kabir et al. [54] showed a decrease in phenolic components and antioxidant activity during refrigerated storage, respectively. It is well known that polyphenols interact with milk proteins to produce insoluble complexes, lowering the total free polyphenols [55].

Textural parameters are a crucial aspect of assessing yogurt quality since customers prefer more consistent, viscous, and cohesive yogurts [56]. The force required to achieve a specific deformation or hardness is a key factor in determining the quality of yogurt; a higher value indicates a firmer sample. Yogurt’s hardness increased with the addition of RFP, and this trend intensified as RFP levels rose. This might be caused by a higher concentration of dry matter and proteins, resulting in a denser structure of yogurt. The values of the textural characteristics varied significantly (*p* < 0.05) over the course of storage. According to Sodini et al. [57], prolonged storage can have an impact on some textural characteristics (firmness), and they suggested that it may be due to a rise in acidity and casein hydration. Cohesiveness is the measure of how well the product withstands a second deformation relative to its resistance under the first deformation [38]. The control sample had the lowest cohesiveness value (0.29), while higher scores were reported in the enriched samples. This may be connected to a stronger gel composition of fortified yogurts. Similar values of textural parameters were reported for yogurts by Yildiz and Ozcan [58]. The gumminess scores of the examined samples demonstrated a generally higher value for YRFP2-produced yogurts. Hardness and cohesiveness lead to gumminess. Yogurt with a high gumminess index also has a high hardness index. The highest amount of adhesiveness was related to control yogurt, while Rhododendron-supplemented (2%) yogurt had the lowest amount of adhesiveness. Adhesiveness decreased as storage time and Rhododendron concentration increased. Our results were consistent with those of the study by Azari-Anpar, Tehrani, et al. [59], which discovered that adding Aloe Vera foliar gel to yogurt samples decreased the adhesiveness of produced samples. According to the texture investigation, the addition of RFP improved the yogurt’s textural qualities proportionately with the concentration.

Because of their antioxidant properties, appealing color, and stability in highly acidic food, anthocyanins are of interest to the food industry [60]. The control sample, as expected, has the greatest L* value, indicating that it has the brightest color, followed by YRFP1 and YRFP2 samples. L* values decreased with the increased incorporation of RFP in the yogurts, demonstrating that the samples darkened as the formulation changed after the addition of a blackish ingredient. Since the a* parameter is related to the redness of the examined sample and the Rhododendron dry flowers added to the yogurts are an anthocyanin-rich product, it was hypothesized that the increase in the a* values would be accompanied by an increase in the incorporation of Rhododendron powder. When grape seed extract was added to yogurt, Yadav et al. [61] observed that the yogurt turned a dark brown or reddish color (L* < 0, a* > 0, and b* < 0) during storage. The samples with higher RFP concentrations also showed higher Chroma values, which measure the intensity of color. The results discussed are connected to sample appearance (Figure 2) and anthocyanin measurement. Regarding its variation during storage, it demonstrated a tendency to increase over time, as seen with a* values. Independent of the amount of fat present, samples containing RFP showed high pigment stability and color. Incorporating RFP into yogurt produces an appealing and anthocyanin-rich product, eliminating the need for artificial coloring. Our findings were consistent with flavored yogurts that also included edible anthocyanin-rich plant materials, including grape pomace [49] and roselle [62] in their composition. Because yogurt has a low pH and encourages the content of anthocyanins, these functional food pigments have been added to it for research purposes.

Sensory evaluations were carried out to ascertain customer preferences for taste, scent, color, and overall quality. Sensory evaluations revealed that the fortified yogurt retained its overall sensory appeal while providing distinctive sour-sweet taste notes. As shown in Figure 3, panelists gave product ratings of between seven and eight (“like moderately” and “like very much”) for each feature. All tested products received positive evaluations from the panelists. The taste, aftertaste, and aroma of the yogurts enriched with RFP were all determined to be acceptable. The unique, dense, and creamy texture of the yogurts was also appreciated. The yogurts’ shades ranged from white pink to white orchid, with YRFP2 being the most white orchid, according to the panelists. The results of the sensory examination point to the 2% powder-added yogurts as having the highest “general acceptability” value. A proper assessment of the percentage of powder addition is necessary to provide uniform physical characteristics and avoid possible textural defects. Higher concentrations may result in a powdery taste; thus, a concentration of 2% RFP is typically suitable for enhancing the textural quality of yogurt. Rhododendron powder is useful for enhancing yogurt’s appealing characteristics and increasing consumer acceptance. As a result, since the panelists did not notice any detrimental effects on the attributes of appearance, color, odor, texture, taste, or overall impression, RFP could be utilized as a yogurt color additive.

## 5. Conclusions

Rhododendron powder has demonstrated its effectiveness as an innovative and appealing component in the production of yogurt. In addition to providing nourishment, Rhododendron is a good source of natural flavors and coloration. The purpose of this study was to assess the functional qualities of RFP-supplemented yogurt that contains Rhododendron dry flowers, a naturally occurring colorant, and antioxidant that is high in health-promoting components such as anthocyanins and polyphenols. The supplemented yogurt samples revealed higher total phenolic contents and antioxidant potential than plain yogurt (control).

Our findings highlight the importance of Rhododendron powder as a rich source of bioactive components with antioxidant activity and suggest incorporating it as a component in fortified yogurt. Based on the sensory evaluations, the developed yogurts with additional powder may be most likely acceptable to consumers due to their generally satisfactory features. The findings of this study show that RFP may offer the food industry a suitable substitute for anthocyanin-rich yogurts. In comparison to the control sample, adding Rhododendron powder to yogurts enhances some nutritional qualities. It provides the dairy sector with new opportunities to meet the rising consumer demand for functional meals by developing innovative, wholesome, and appealing yogurt products. To fully exploit the potential of this fortified yogurt product, additional research on formulation optimization and storage stability is required.

Moreover, with these results, additional research is required to estimate the precise number of bioactive compounds absorbed by the human body while considering the constraints of in vitro research. Additionally, a thorough understanding of the interactions between food matrices and biologically active substances like polyphenols is necessary to develop products with additional health benefits.

## Figures and Tables

**Table 1:** Phytochemical content of the RFP extract.

Parameters	Extract
Total anthocyanins (mg C3G/g DW)	1.05 ± 0.24
Total flavonoids (mg CE/g DW)	8.58 ± 0.19
Total polyphenols (mg GAE/g DW)	21.46 ± 0.63
DPPH (µmol TE/g DW)	16.89 ± 0.22
L*	42.82 ± 0.25
a*	12.53 ± 0.07
b*	3.25 ± 0.09
Chroma	12.94 ± 0.08
Hue angle	0.25 ± 0.01
ΔE	44.73 ± 0.26

**Table 2:** Chemical composition and storage stability of the control and RFP enriched yogurts.

Component (%)	Sample	Storage Period (Days)		
		1	14	28
Moisture	YC	86.77 ± 0.17 ^xA^	86.82 ± 0.12 ^xA^	86.77 ± 0.17 ^xA^
	YRFP1	84.48 ± 0.69 ^yA^	84.85 ± 0.69 ^yA^	85.10 ± 0.69 ^yA^
	YRFP2	82.58 ± 0.69 ^zA^	82.49 ± 0.69 ^zA^	82.23 ± 0.00 ^zA^
Fat	YC	3.85 ± 0.03 ^yA^	3.85 ± 0.03 ^xyA^	3.85 ± 0.03 ^xA^
	YRFP1	3.88 ± 0.05 ^xyA^	3.81 ± 0.05 ^yAB^	3.77 ± 0.05 ^yB^
	YRFP2	3.91 ± 0.05 ^xA^	3.89 ± 0.05 ^xAB^	3.85 ± 0.05 ^xB^
Total protein	YC	3.70 ± 0.09 ^xyA^	3.70 ± 0.09 ^yA^	3.70 ± 0.09 ^xyA^
	YRFP1	3.74 ± 0.01 ^yA^	3.69 ± 0.01 ^xyA^	3.67 ± 0.01 ^yA^
	YRFP2	3.81 ± 0.02 ^xA^	3.78 ± 0.02 ^xA^	3.76 ± 0.02 ^xA^
Ash	YC	0.74 ± 0.07 ^zA^	0.74 ± 0.07 ^zA^	0.74 ± 0.07 ^zA^
	YRFP1	0.88 ± 0.03 ^yA^	0.87 ± 0.03 ^yA^	0.86 ± 0.03 ^yA^
	YRFP2	0.99 ± 0.03 ^xA^	0.98 ± 0.03 ^xA^	0.97 ± 0.03 ^xA^
Crude fiber	YC	0.00 ± 0.00 ^zA^	0.00 ± 0.00 ^zA^	0.00 ± 0.00 ^zA^
	YRFP1	0.94 ± 0.03 ^yA^	0.93 ± 0.03 ^yAB^	0.92 ± 0.03 ^yB^
	YRFP2	1.10 ± 0.01 ^xA^	1.12 ± 0.01 ^xA^	1.10 ± 0.00 ^xA^
Energy value (kcal 100 g^−1^ FW)	YC	84.00 ± 0.50 ^zA^	83.79 ± 0.39 ^zA^	84.00 ± 0.50 ^xyA^
	YRFP1	89.13 ± 2.58 ^yA^	87.18 ± 2.58 ^yAB^	86.00 ± 2.60 ^yB^
	YRFP2	96.12 ± 2.73 ^xA^	96.22 ± 2.73 ^xA^	97.10 ± 0.30 ^xA^
CHO	YC	4.93 ± 0.12 ^zA^	4.88 ± 0.10 ^zA^	4.94 ± 0.12 ^zA^
	YRFP1	6.07 ± 0.30 ^yA^	5.84 ± 0.30 ^yA^	5.67 ± 0.31 ^yA^
	YRFP2	7.60 ± 0.30 ^xA^	7.73 ± 0.31 ^xA^	8.08 ± 0.05 ^xA^
pH	YC	4.62 ± 0.11 ^xA^	4.51 ± 0.01 ^xA^	4.46 ± 0.01 ^xA^
	YRFP1	4.56 ± 0.01 ^xyA^	4.36 ± 0.01 ^yB^	4.09 ± 0.02 ^yC^
	YRFP2	7.60 ± 0.30 ^yA^	7.73 ± 0.30 ^zB^	8.08 ± 0.02 ^zC^

FW–fresh weight, averages with different letters “A”, “B”, “C” in the rows and “x”, “y”, “z” in the columns signify statistically significant differences (*p* < 0.05).

**Table 3:** Phytochemical profile of the control and supplemented yogurt and stability during 28 days of storage.

Parameters	Sample	Storage Period (Days)		
		1	14	28
Total polyphenolic compounds (mg GAE/100 g DW)	YC	4.25 ± 0.50 ^zA^	3.82 ± 0.26 ^zA^	3.09 ± 0.57 ^zA^
	YRFP1	11.99 ± 0.74 ^yA^	10.74 ± 0.68 ^yA^	9.49 ± 0.91 ^yA^
	YRFP2	16.42 ± 8.74 ^xA^	19.77 ± 0.93 ^xA^	18.84 ± 1.27 ^xA^
Total flavonoids (mg CE/100 g DW)	YC	0.00 ± 0.00 ^zA^	0.00 ± 0.00 ^zA^	0.00 ± 0.00 ^zA^
	YRFP1	1.20 ± 0.26 ^yA^	0.87 ± 0.27 ^yAB^	0.57 ± 0.42 ^xyB^
	YRFP2	1.85 ± 0.29 ^xA^	1.42 ± 0.43 ^xB^	0.87 ± 0.26 ^xC^
Total anthocyanins (mg/100 g DW)	YC	0.00 ± 0.00 ^zA^	0.00 ± 0.00 ^zA^	0.00 ± 0.00 ^zA^
	YRFP1	1.46 ± 0.64 ^yA^	1.05 ± 0.01 ^yAB^	0.59 ± 0.28 ^yB^
	YRFP2	2.65 ± 0.72 ^xA^	2.11 ± 0.05 ^xAB^	1.78 ± 0.10 ^xB^
DPPH (μmol TE/100 g)	YC	3.20 ± 0.21 ^zA^	2.24 ± 0.53 ^zB^	1.08 ± 0.41 ^zC^
	YRFP1	9.41 ± 0.42 ^yA^	8.72 ± 0.68 ^yAB^	7.76 ± 0.32 ^yB^
	YRFP2	15.89 ± 0.91 ^xA^	14.72 ± 0.73 ^xB^	13.48 ± 0.84 ^xC^

Averages with different letters “A”, “B”, “C” in the rows and “x”, “y”, “z” in the columns signify statistically significant differences (*p* < 0.05).

**Table 4:** Texture of yogurts supplemented with RFP after 1, 14, and 28 days of storage.

Textural Parameters	Sample	Storage Period (Days)		
		1	14	28
Cohesiveness	YC	0.29 ± 0.00 ^yA^	0.31 ± 0.00 ^zAB^	0.32 ± 0.00 ^yB^
	YRFP1	0.33 ± 0.01 ^xyA^	0.34 ± 0.01 ^yAB^	0.37 ± 0.01 ^xyB^
	YRFP2	0.34 ± 0.01 ^xA^	0.36 ± 0.01 ^xB^	0.38 ± 0.01 ^xC^
Springiness	YC	0.50 ± 0.02 ^xA^	0.51 ± 0.02 ^xAB^	0.53 ± 0.02 ^xB^
	YRFP1	0.30 ± 0.01 ^yA^	0.33 ± 0.01 ^yB^	0.36 ± 0.01 ^yC^
	YRFP2	0.24 ± 0.01 ^zA^	0.29 ± 0.00 ^zB^	0.31 ± 0.00 ^zC^
Hardness, N	YC	5.65 ± 0.00 ^zA^	5.66 ± 0.00 ^zA^	5.66 ± 0.00 ^zA^
	YRFP1	7.50 ± 0.02 ^yA^	7.52 ± 0.02 ^yAB^	7.53 ± 0.02 ^yB^
	YRFP2	11.59 ± 0.02 ^xA^	11.61 ± 0.02 ^xAB^	11.63 ± 0.02 ^xB^
Gumminess, N	YC	1.63 ± 0.01 ^zA^	1.65 ± 0.01 ^zAB^	1.66 ± 0.01 ^zB^
	YRFP1	2.74 ± 0.02 ^yA^	2.75 ± 0.02 ^yAB^	2.76 ± 0.02 ^yB^
	YRFP2	4.08 ± 0.01 ^xA^	4.10 ± 0.01 ^xAB^	4.11 ± 0.01 ^xB^
Adhesiveness, mJ	YC	−2.51 ± 0.01 ^zA^	−2.53 ± 0.01 ^zAB^	−2.55 ± 0.01 ^xB^
	YRFP1	−6.72 ± 0.03 ^yA^	−6.74 ± 0.03 ^yAB^	−6.75 ± 0.03 ^yB^
	YRFP2	−11.42 ± 0.02 ^xA^	−11.45 ± 0.02 ^xAB^	−11.47 ± 0.02 ^xB^

Averages with different letters “A”, “B”, “C” in the rows and “x”, “y”, “z” in the columns signify statistically significant differences (*p* < 0.05).

**Table 5:** Color evaluation of yogurt samples and stability during 28 days of storage.

Parameters	Sample	Storage Period (Days)		
		1	14	28
L*	YC	96.97 ± 0.26 ^xA^	96.40 ± 0.26 ^xAB^	95.98 ± 0.26 ^xB^
	YRFP1	82.49 ± 0.72 ^yA^	81.29 ± 0.72 ^yB^	80.24 ± 0.72 ^yC^
	YRFP2	71.54 ± 0.96 ^zA^	70.84 ± 0.96 ^zAB^	70.46 ± 0.96 ^zB^
a*	YC	−9.22 ± 0.09 ^zA^	−8.75 ± 0.09 ^zB^	−8.49 ± 0.09 ^zC^
	YRFP1	6.23 ± 0.12 ^yA^	8.83 ± 0.12 ^yB^	10.53 ± 0.12 ^yC^
	YRFP2	12.14 ± 0.11 ^xA^	14.89 ± 0.11 ^xAB^	16.80 ± 0.11 ^xB^
b*	YC	17.55 ± 0.21 ^zA^	17.83 ± 0.21 ^zB^	18.21 ± 0.21 ^zC^
	YRFP1	29.47 ± 0.26 ^yA^	29.77 ± 0.26 ^yB^	30.05 ± 0.26 ^yC^
	YRFP2	41.05 ± 0.16 ^xA^	41.24 ± 0.16 ^xAB^	41.33 ± 0.16 ^xB^
Chroma	YC	19.83 ± 0.22 ^zA^	19.86 ± 0.22 ^zA^	20.09 ± 0.22 ^zA^
	YRFP1	30.12 ± 0.25 ^yA^	31.05 ± 0.25 ^yAB^	31.84 ± 0.25 ^yB^
	YRFP2	42.81 ± 0.17 ^xA^	43.84 ± 0.17 ^xA^	44.61 ± 0.17 ^xA^
Hue angle	YC	178.91 ± 0.00 ^xA^	178.89 ± 0.00 ^xB^	178.87 ± 0.00 ^xC^
	YRFP1	1.36 ± 0.00 ^yA^	1.28 ± 0.00 ^yB^	1.23 ± 0.00 ^yC^
	YRFP2	1.28 ± 0.00 ^zA^	1.22 ± 0.00 ^zAB^	1.18 ± 0.00 ^zB^

Averages with different letters “A”, “B”, “C” in the rows and “x”, “y”, “z” in the columns signify statistically significant differences (*p* < 0.05).

## Data Availability

Data is contained within the article.
